# Effects of horizontal compared to vertical-based plyometric jump training on semi-professional soccer player's performance

**DOI:** 10.1038/s41598-023-37213-x

**Published:** 2023-06-20

**Authors:** Hadi Nobari, Filipe Manuel Clemente, Norodin Vali, Ana Filipa Silva, Daniel van den Hoek, Rodrigo Ramirez-Campillo

**Affiliations:** 1 Department of Exercise Physiology, Faculty of Educational Sciences and Psychology, University of Mohaghegh Ardabili, Ardabil, 5619911367 Iran; 2 Faculty of Sport Sciences, University of Extremadura, 10003 Cáceres, Spain; 3 Escola Superior Desporto e Lazer, Instituto Politécnico de Viana do Castelo, Rua Escola Industrial e Comercial de Nun’Álvares, 4900-347 Viana do Castelo, Portugal; 4 Instituto de Telecomunicações, Delegação da Covilhã, 1049-001 Lisbon, Portugal; 5 Department of Exercise Physiology, Shahid Rajaee Teacher Training University, Tehran, Iran; 6 Research Center in Sports Performance, Recreation, Innovation and Technology (SPRINT), 4960-320 Melgaço, Portugal; 7 The Research Centre in Sports Sciences, Health Sciences and Human Development (CIDESD), 5001-801 Vila Real, Portugal; 8 University of the Sunshine Coast, School of Health and Behavioural Sciences, Sippy Downs, QLD Australia; 9 Exercise and Rehabilitation Sciences Laboratory, School of Physical Therapy, Faculty of Rehabilitation Sciences, Universidad Andres Bello, Santiago, Chile

**Keywords:** Physiology, Medical research, Engineering

## Abstract

This study aimed to compare the effects of horizontal (HJ) and vertical (VJ)-based plyometric jump training on male semi-professional soccer player's performance (e.g., change-of-direction speed [5-0-5 test]; 10-m, 20-m, and 30-m linear sprint speed). A parallel-study design was conducted. Participants were organized into HJ (n = 10) or VJ (n = 9) during 12 weeks. Measures of athletic performance were obtained in four phases: (i) before and (ii) end of the pre-season, (iii) during (weeks 7th), and (iv) after the intervention. The within-group analysis revealed that both HJ and VJ improved change of direction (${x}^{2}$ = 27.783; p < 0.001 (${x}^{2}$ = 21.635; p < 0.001),), 10-m linear sprint time (${x}^{2}$ = 28.576; p < 0.001), 20-m linear sprint time (${x}^{2}$ = 28.969, p < 0.001), and 30-m linear sprint time (${x}^{2}$ = 26.143; p < 0.001). Similarly, the VJ-group also imposed significant changes on 5-0-5 time, 10-m linear sprint time (${x}^{2}$ = 25.787; p < 0.001), 20-m linear sprint time (${x}^{2}$ = 24.333, p < 0.001), and 30-m linear sprint time (${x}^{2}$ = 22.919; p < 0.001). Between-group analysis revealed no significant differences in any of the assessment moments. HJ and VJ plyometric jump training are effective for improving the change-of-direction and a linear sprint of semi-professional players with no difference between types of intervention.

## Introduction

Soccer is characterized by its intermittence in terms of physiological and locomotor demands^1^. A prevalence of low-to-moderate running is interspersed by periods of high-intensity to all-out efforts characterized by repeated sprints, peak speed, or high-intensity running (e.g., up to 40 actions at > 21 km/h may be attained in the match)^2^. From the typical 9–14 km covered by male soccer players, 1000 to 1500 m can be covered at high-intensity running^3^. Moreover, over a soccer match, up to 1400 other actions, including up to 700 directional changes and 600 accelerations and decelerations have been reported^2,4^

Coping with the physical demands of a soccer match requires players to have well-developed physical fitness. Straight-line sprinting and change-of-direction (COD) are soccer's most recurrent actions preceding goals^5^. However, there are significant relationships between aerobic fitness, match-running performance^6,7^, and other qualities such as sprinting, COD, or lower limb power^8,9^.

Providing an adequate stimulus for improving different physical qualities in soccer players is a complex challenge since performance is multi-dependent, and more than one physical quality requires stimulation^10^. One of the targets is improving lower-body power by using different strength training methods. Plyometric jump training (PJT) has gained popularity and is effective for enhancing sprinting performance^11^, vertical jump^12^, change-of-direction^13–15^, and running economy^16^. PJT uses jumps to stress the musculotendinous unit^17^, using different directional and types of jumps and hops^18^.

One of the debates about the PJT implementation is related to the direction of jumps. The hypothesis of vertical and horizontal force application producing different effects on physical fitness adaptations has been a focus of research^19^. It is hypothesized that muscle action velocity or movement direction relates to specificity in the adaptations of neural mechanisms involved in force production^20^. For example, an experimental study comparing only vertical vs. horizontal vs. combined PJT on soccer players revealed that these training interventions significantly improved explosive actions, balance, and intermittent endurance capacity^21^. Interestingly, a meta-analysis comparing horizontal jump (HJ) and vertical jump (VJ) training reported that both effectively improve performance in horizontal and vertical directions. However, HJ appears more effective as it has an equivalent effect on vertical performance and a more significant impact on HJ performance than VJ^19^.

Although research comparing the effects of HJ and VJ-based PJT has reported their impact on vertical and horizontal jumping performance, the meta-analysis summarized only nine studies^19^ and four conducted in soccer players^21–24^. These four original works focused on identifying the effects of plyometric programs on jumping and sprinting. In contrast, only one focused on endurance performance^21^. Two studies lasted 8 weeks^22,24^, one study lasted three weeks^23^, and one study lasted six weeks^21^. Thus, the body of knowledge about vertical vs. horizontal PJT on soccer players is small, and the interventions are short to moderate duration in time. Therefore, further research should be conducted to generalize the findings and, in particular, extend the intervention over the season while considering it as part of the team’s periodization. Thus, this study aimed to compare the effects of HJ and VJ-based plyometric jump training on COD and linear sprint for soccer players. Based on the objective, the hypothesis is that HJ may play a greater role in improving COD and linear sprinting while considering the force vector.

## Methods

### Study design

The current study followed an experimental parallel-group design. The research protocol was approved by ethics committee of the University of Mohaghegh Ardabili approved the research protocol with code 16.05.2020. All participants were informed of study procedures and the use of data before providing informed consent. In this project, we have followed all the Helsinki guidelines at all stages for human studies.

### Setting

**Figure 1:**
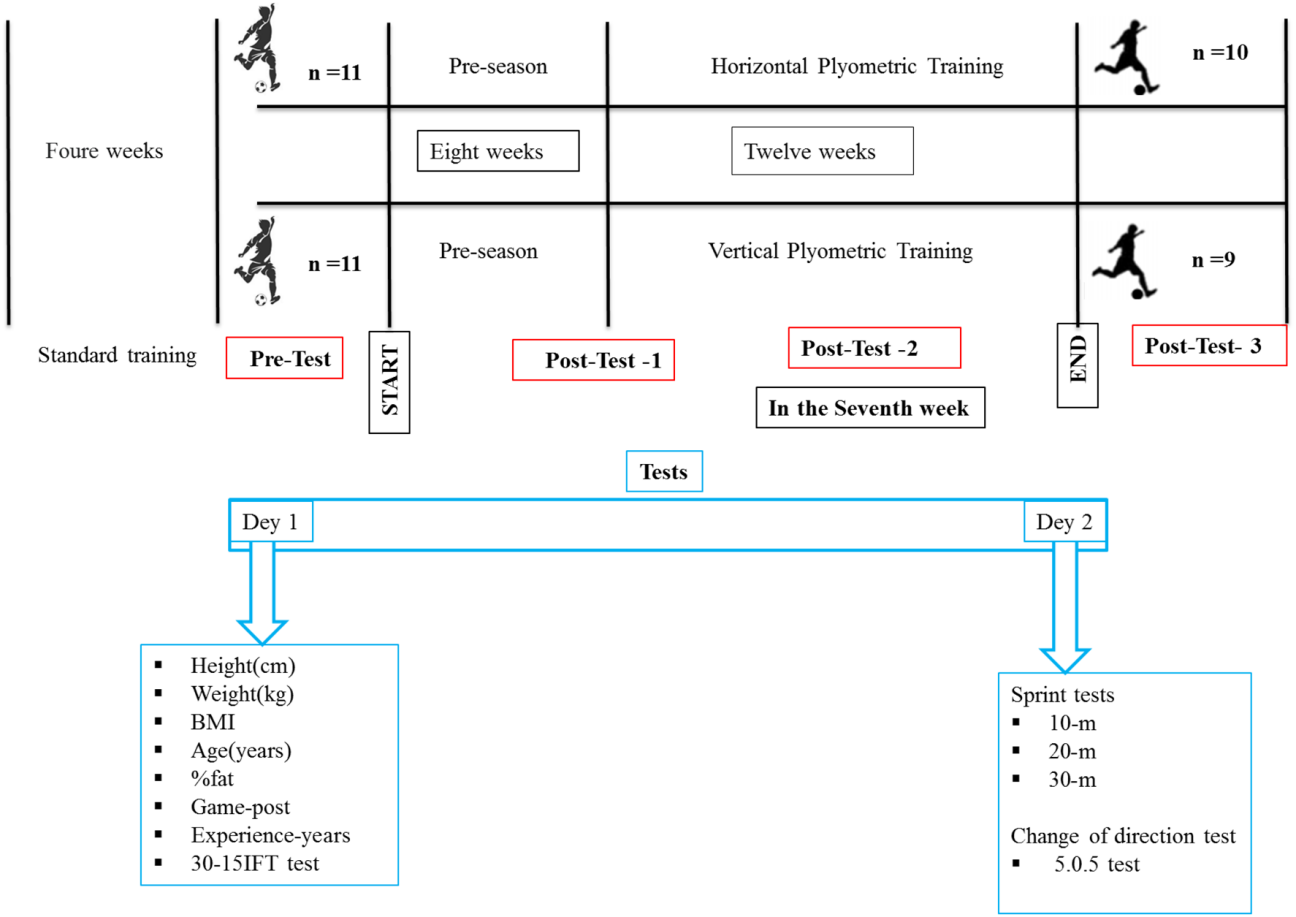
Study's timeline.

Data collection occurred from 21st August 2020–31st January 2021. For context, the study began after four weeks of pre-season commencement. The training intervention lasted 21 weeks and we present the study's timeline in Fig. 1. Players were assessed 4 times over the period. Before each assessment, 72 h of rest were guaranteed regarding the last training session/match. The assessments were always performed on the same day of the week (change-of-direction and linear sprint test on Saturday and V_IFT_ test on Monday). The assessments started at 10 a.m.–12 p.m. The first assessment's average temperature and relative humidity were 31 °C and 2%, respectively. The second assessment's average temperature and relative humidity were 13 °C and 25%, respectively. The third assessment's average temperature and relative humidity were 12 °C and 40%, respectively. The fourth assessment’s average temperature and relative humidity were 16 °C and 35%, respectively. Assessments occurred in full sun. The training interventions occurred once per week.

### Context of training intervention

**Figure 2:**
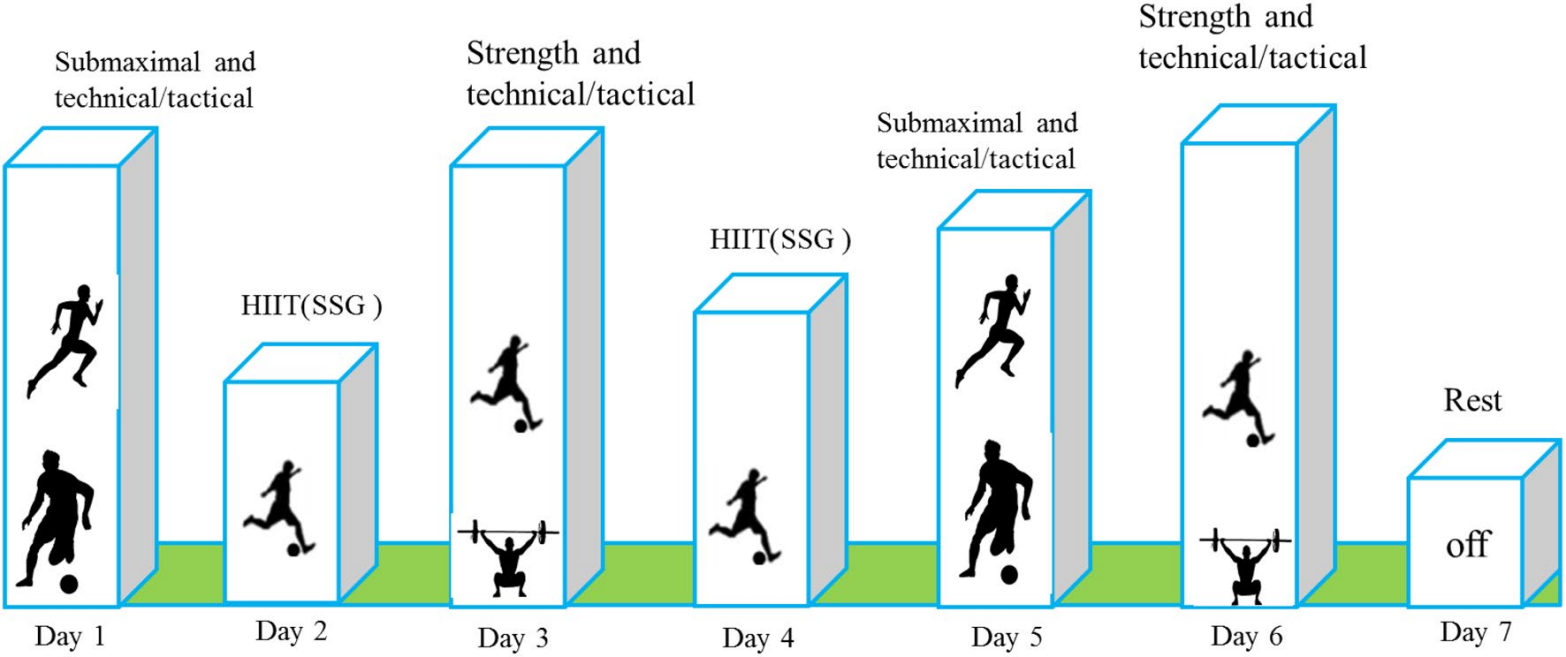
Details of habitual training routine performed by both groups over the 8-week pre-season.

The pre-season consisted of six sessions per week for eight weeks. Microcycles of weekly training are presented in Fig. 2. In the pre-season, most training sessions included (in this order): submaximal aerobic training (65–75% HR_max_), small-sided game (80–95%HRmax), high/ medium interval training (according to the results of V_IFT_, this volume was used 30-30, 15-15, 10-10. Also the intensity of exercise is selected in the range between 70 to 95% V_IFT_) and strength training. The strength training performed in the pre-season was circuit-based and was the same for all players. It consisted of 8 stations: the order of the stations was as follows: squats (10 reps), push up (20 reps), flat plank (25 s), step-up (8 reps), barbell shoulder press (8 reps), burpee (7 reps), side plank (20 reps) and bicycle crunches (30 reps). The rest between each station was 90 s, and each player performed each station thrice. In addition to regular training, players participated in five friendly games during the pre-season.

**Figure 3:**
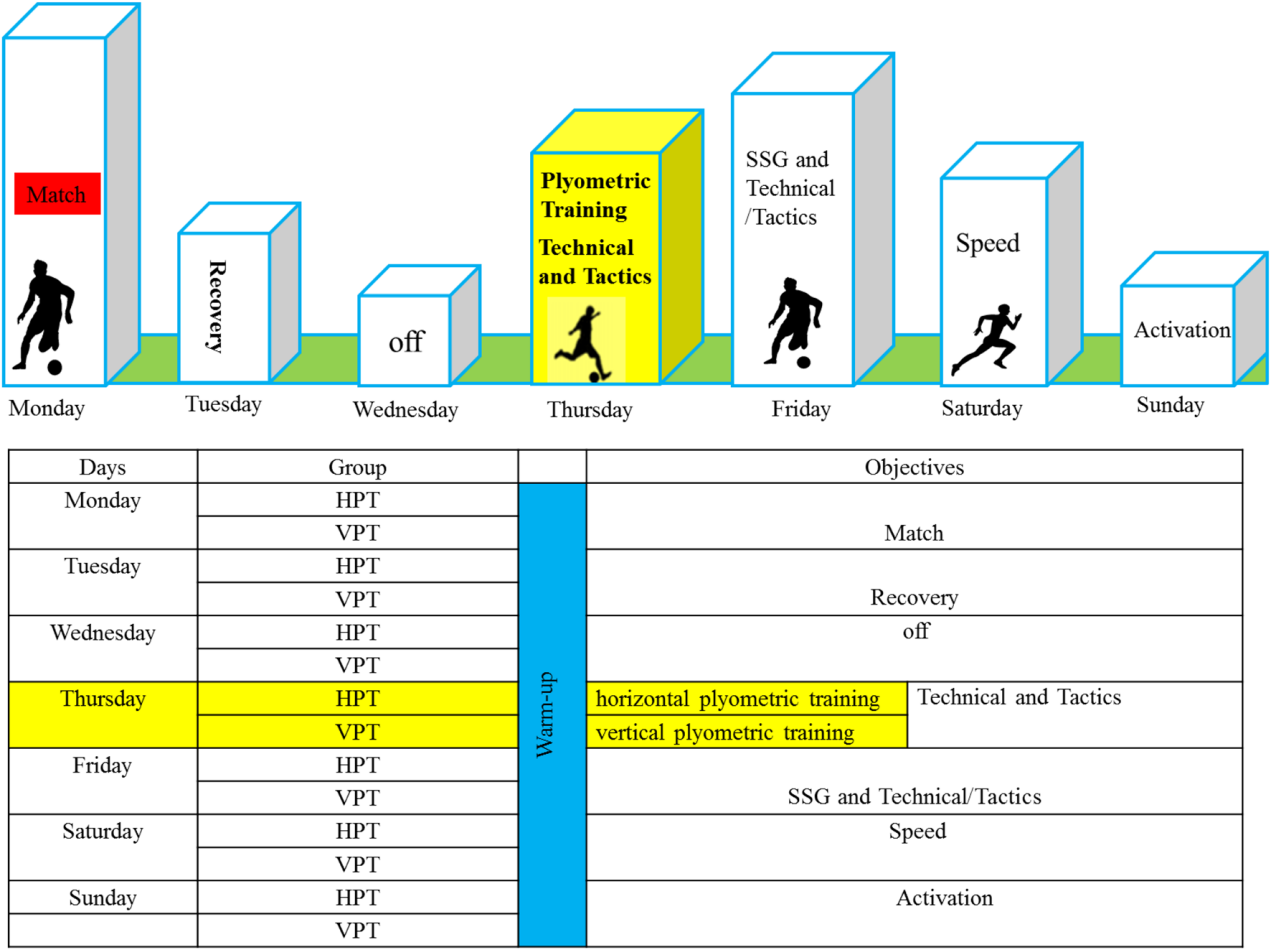
Details of habitual training routine performed by both groups over the 13-week study.

During the season, participants trained five times per week, along with one match. Figure 3 shows the details of each training session during the in-season period and the scheduling of the research protocol within the training schedule.

### Participants

**Figure 4:**
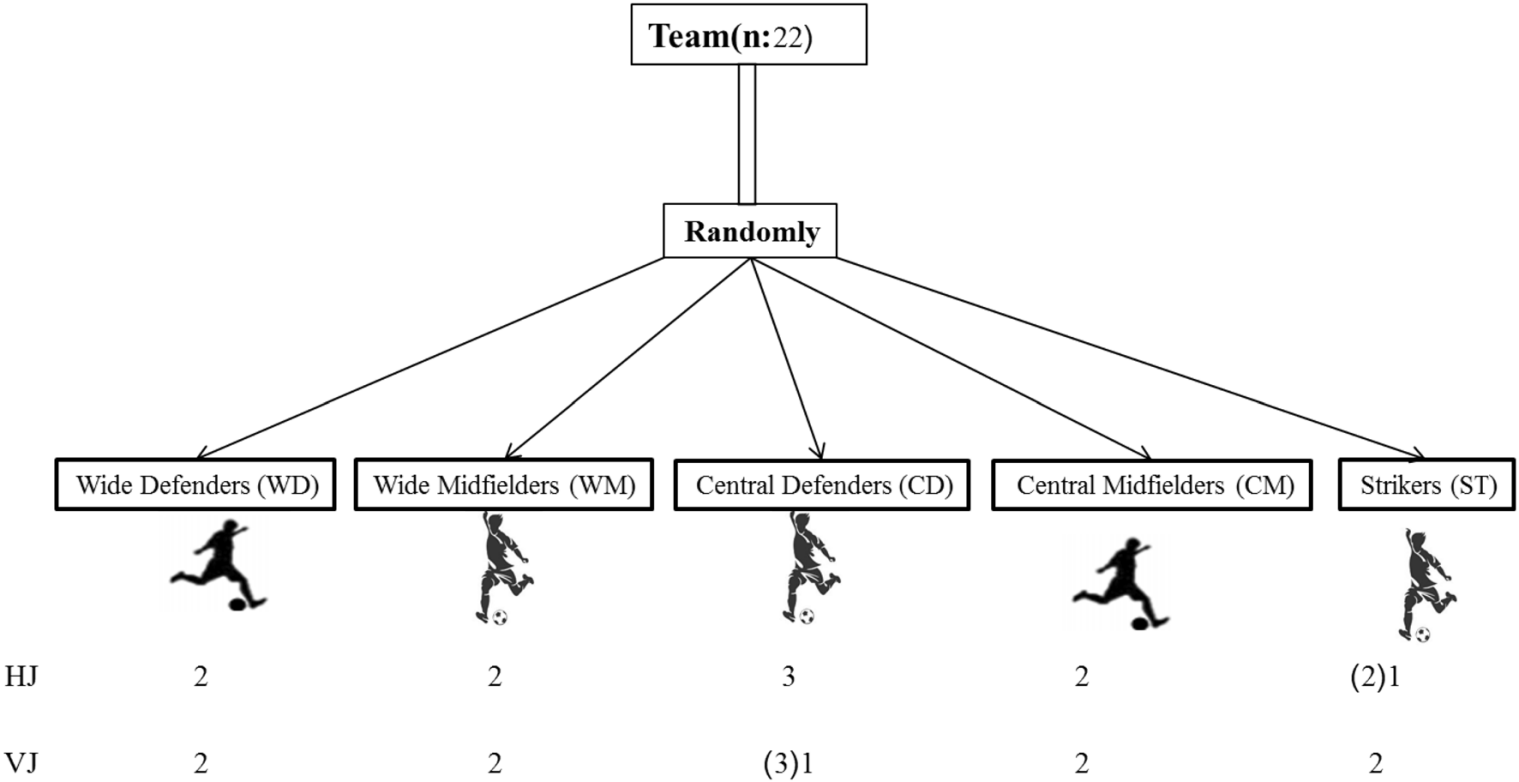
CONSORT chart of study and analysis of participants. HJ: horizontal jump; VJ: vertical jump; (2) 1: It means removing a player from the study for analysis; (3) 1: It means removing two players from the study for analysis.

Twenty-three semi-professional soccer players (age: 23.1 ± 2.8 years, height: 178.3 ± 4.8 cm, body mass: 72.4 ± 4.9 kg and BMI: 22.7 ± 1.4 kg/m^2^) all from the same team participated in the study (Table 1). Subjects participated in the 8-week pre-season jointly and then, 1 week before the start of the season, were divided randomly into two training groups: the unloaded horizontal plyometric training group (HPT; n = 11) and the unloaded vertical plyometric training group (VPT; n = 11). The exclusion criteria were injuries and inability to participate in three consecutive training sessions. Due to injuries or missed sessions, were eliminated three players from the study (e.i., two players from the VPT and one from the HPT). As a result, 19 players (HPT: 10n and VPT: 9n; see Fig. 4) finished the research.

**Table 1:** Physical characteristics (mean-SD) of the participants.

Group	Participants (N)	Age (years)	Experience (years)	Height (cm)	Body mass (kg)	BMI (kg/m2)	Defenders (N)	Midfielders (N)	Attackers (N)
HJ	10	23.33 ± 2.9	11.77 ± 1.8	179.22 ± 3.7	70.11 ± 4.9	21.83 ± 1.6	5	4	1
VJ	9	23.30 ± 2.9	11.60 ± 2.1	178.00 ± 5.8	72.40 ± 4.8	22.84 ± 1.0	3	4	2

*HJ* horizontal-based jump group, *VJ* vertical-based jump group, *BMI* body mass index, *N* number.

### Experimental approach

This study was conducted in four phases. All tests (e.g., 10, 20, and 30 m sprint testing, 5-0-5 agility test, anthropometric testing, and 15–30_VIFT_) only included in phases one and two were performed in each stage. Results of the 15–30_VIFT_ were used to match players of similar abilities, who were then randomized into either HJ or VJ groups. Phase one occurred one week before the start of the pre-season. Then stage two was performed one week before the beginning of the competition season. Afterwards, phase three commenced at the mid-point of the competitive season. The seven-week PJT intervention began in this phase. Finally, phase four testing was performed three days after the season's final match.

Two separate warm-up protocols were performed in this study. The first warm-up protocol (e.i., FIFA 11+ warm-up), before running the evaluation tests has been done in each stage (details of this warm-up protocol are given in Table 2). The second warm-up protocol every week was provided only on the day when plyometric exercises were performed. The details of this warm-up protocol are shown in Table 3.

**Table 2:** Exercise programs performed in FIFA 11+ warm-up.

No.	Section I: Running movements (8–10 min)	
1	Straight ahead	5 min
2	Hip out	10 reps
3	Hip in	10 reps
4	Circling partner	5 reps
5	Shoulder contact	6 reps
6	Quick forwards or backwards	7 reps
	Section II: Plyometric, strength, and balance movements (10–12 min)	
7	Plank with alternating leg	3 set × 20 s
8	Sideway plank with leg lift	3 set × 15 s
9	Nordic hamstring curl	2 set × 10 reps
10	Single leg stance	2 set × 30 s
11	Walking lunges	2 set × 8 reps
12	One leg squats	1 set × 8 reps
13	Vertical jumps	2 set × 6 reps
14	Lateral jumps	2 set × 6 reps
	Section III: Running ABC (2–3 min)	
15	Running across the pitch	Approximately with 70–80% maximum pace × 3 reps
16	Running bounding	2 set × 16 reps
17	Running plant or cut	2 set × 4 step

**Table 3:** The warm-up protocol before performing each plyometric training session.

No	Section I: Running movements (8–10 min)	
1	Straight ahead	5 min
2	Hip out	10 reps
3	Hip in	10 reps
4	Circling partner	5 reps
5	Shoulder contact	6 reps
6	Quick forwards or backwards	7 reps
	Section II: Plyometric, strength, and balance movements (10–12 min)	
7	Flat plank	3 set × 20 s
8	Sideway plank with leg lift	3 set × 15 s
9	Walking lunges	2 set × 8 reps
10	One leg squats	1 set × 8 reps
	Section III: Running ABC (2–3 min)	
11	Running across the pitch	Approximately with 70–80% maximum pace × 3 reps
12	Running bounding	2 set × 16 reps
13	Running plant or cut	2 set × 4 step
	Section IV: Skills (5–7 min)	
14	Passing and moving	2 min
15	Pass and a one-two	2 min
16	Pass and one-three	2 min

### Details of the plyometric training program

Prior to the competitive season (August to October), all subjects participated in an eight-week pre-season that included six training sessions per week. During the competitive season (October to January), the subjects exercise 5 sessions per week, participating in an official football match every Monday. Each group performed its plyometric training protocol once a week for 13 weeks. Details of the plyometric exercise protocol are given in Table 2. Training sessions were always supervised by a strength and conditioning coach. Verbal encouragement was used to perform the movements in both groups equally. All training sessions were held on natural grass with stock shoes and soccer training uniforms. The pattern of formation of the names of movements is by the study of Nicholas^20^, but in this study, the intensity, rest time, the number of repetitions and duration of training intervention vary.

The total number of contacts per player was 912, which was considered equal for both groups. Movement intensity, volume and speed were considered the same during the research period (Table 4).

**Table 4:** Training volume during the vertical and horizontal plyometric training programs.

Group	Exercises	Weeks 1–13 sets	Rest	Speed	Contacts
HPT	Horizontal ankle jumps	4	1–2 min	Explosive	6
	Long jumps	4	1–2 min	Explosive	6
	Diagonal obstacle jumps	4	1–2 min	Explosive	7
Total volume		12			19
VPT	Vertical ankle jumps	4	1–2 min	Explosive	6
	Countermovement jump	4	1–2 min	Explosive	6
	Squat jump	4	1–2 min	Explosive	7
Total volume		12			19

Intensity: exercises were performed with maximal effort (intensity: 100%), Rest: 1–2 min between sets and 2–3 min between exercises.

*HPT* horizontal plyometric training, *VPT* vertical plyometric training.

### Testing schedule

Definitive tests were performed in a fixed order over two days. On the first test day, anthropometric measurements were made, followed by V_IFT_ test. The second day was devoted to change-of-direction and linear sprint tests.

### Anthropometry

The height and body mass were assessed at 10–12 a.m. after waking. The same observer made the assessments. The height was evaluated using a stadiometer (Seca 217 Stable stadiometer, Hamburg, Germany), with players wearing shorts, t-shirts, and no shoes. The same for body mass. The same observer on the right side of the body made all the measures. The outcome of the height assessment was the height in cm, for the body mass was the mass in kg.

### Sprint test

A sprint linear test of three different distances at 10-m, 20-m, and 30-m was used to determine the sprinting time of the players. The sprint tests started in a split position of the foot, always with the same preferable leg for the player. The starting point position was 70 cm behind the first photocells marking the starting line^25^. Four pairs of photocells were used (starting line, 10-m, 20-m and 30-m). The height of the photocells (Newtest Powertimer 300-series testing system, Finland) was positioned based on the player’s hips. Players performed two trials, interspaced by 120 s of rest. The smallest time (s) to complete each linear sprint test was used for further data treatment. The intra-class correlation of these tests was between 0.81 and 0.87 and coefficient of variation was 1.1–1.4%.

### Change-of-direction test

The modified 5-0-5 test protocol was employed to measure the players' COD time and COD deficit. The test consists of starting in a standing position (foot split) and accelerating over a 10-m distance before performing two COD of 180º (from A to C point was 5 + 5 m). The time from the final 10-m (5 + 5 m) is recorded using two pairs of photocells (Newtest Power timer 300-series testing system, Finland). The photocell height was adjusted based on the height of the player’s hip. The players were allowed to use the preferred leg for braking and turning movements. However, they were always asked to use the same leg. The same instruction was used for the foot in front at the starting position. Each participant performed two trials, with a rest period of three minutes. The COD time (s) was obtained for each trial. The smallest time was used for further statistical procedures^26^. The COD deficit was used by subtracting the COD time by the linear speed time at 10-m^27^. The intra-class correlation of this test was 0.86 and coefficient of variation was 1.9%.

### Statistical procedures

The descriptive statistics of the current study are presented in the form of mean and standard deviation. Due to the small sample and the violation of the normality assumption (p < 0.05), we have used non-parametric tests to analyze variation. In this case, we have used the Friedman test for within-group changes analysis (comparison between time points). The pots-hoc analysis for this case was performed using the Wilcoxon signed-rank test, which is indicated for being more conservative and having lower power^28^. Additionally, the between-group differences were tested using the Mann–Whitney U. The statistical procedures were executed using SPSS statistical software (version 28.0.0.0, IBM, USA). An alpha level of p < 0.05 was adopted for all tests.

### Institutional review board statement

The University of Mohaghegh Ardabili approved the research protocol with code 16.05.2020. All participants were informed of study procedures and the use of data prior to providing informed consent.

## Results

The descriptive statistics of physical fitness outcomes of both training groups in the four-time points of assessment can be found in Table 5. The within-group analysis revealed that HJ-group imposed significant changes on 5-0-5 time (${x}^{2}$ = 27.783; p < 0.001), CODdeficit (${x}^{2}$ = 17.505; p < 0.001), 10-m linear sprint time (${x}^{2}$ = 28.576; p < 0.001), 20-m linear sprint time (${x}^{2}$ = 28.969, p < 0.001), and 30-m linear sprint time (${x}^{2}$ = 26.143; p < 0.001). Similarly, the VJ-group also imposed significant changes on 5-0-5 time (${x}^{2}$ = 21.635; p < 0.001), CODdeficit (${x}^{2}$ = 8.159; p = 0.043), 10-m linear sprint time (${x}^{2}$ = 25.787; p < 0.001), 20-m linear sprint time (${x}^{2}$ = 24.333, p < 0.001), and 30-m linear sprint time (${x}^{2}$ = 22.919; p < 0.001). Between-group analysis revealed no significant differences in any of the assessment moments.

**Table 5:** Descriptive statistics (mean and standard deviation) of physical fitness outcomes in the four assessment moments.

Outcome	HJ	HJ	HJ	HJ	HJ	VJ	VJ	VJ	VJ	VJ	Between-group (A1)	Between-group (A2)	Between-group (A3)	Between-group (A4)
	A1	A2	A3	A4	Within-group	A1	A2	A3	A4	Within-group				
5-0-5 (s)	2.36 ± 0.05 ^b: p = 0.005|Δ1.7%^ ^c: p = 0.005|Δ2.5%^ ^d: p = 0.005|Δ2.5%^	2.32 ± 0.04 ^a: p = 0.005|Δ1.7%^ ^c: p = 0.017|Δ0.9%^ ^d: p = 0.007|Δ0.9%^	2.30 ± 0.04 ^a: p = 0.005|Δ2.5%^ ^b: p = 0.005|Δ0.9%^	2.30 ± 0.03 ^a: p = 0.005|Δ2.5%^ ^b: p = 0.007|Δ0.9%^	${x}^{2}$ = 27.783 p < 0.001	2.35 ± 0.04 ^b: p = 0.007|Δ1.3%^ ^c: p = 0.008|Δ2.1%^ ^d: p = 0.008|Δ2.1%^	2.32 ± 0.33 ^a: p = 0.007|Δ1.3%^ ^c: p = 0.016|Δ0.9%^ ^d: p = 0.028|Δ0.9%^	2.30 ± 0.03 ^a: p = 0.008|Δ2.1%^ ^b: p = 0.016|Δ0.9%^	2.30 ± 0.03 ^a: p = 0.008|Δ2.1%^ ^b: p = 0.028|Δ0.9%^	${x}^{2}$ = 21.635 p < 0.001	Z = − 0.452 p = 0.651	Z = − 0.082 p = 0.934	Z = − 0.993 p = 0.321	Z = − 0.290 p = 0.772
COD deficit (s)	0.46 ± 0.05 ^b: p = 0.023|Δ1.7%^ ^c: p = 0.011|Δ2.5%^ ^d: p = 0.008|Δ2.5%^	0.48 ± 0.05 ^a: p = 0.023|Δ1.7%^ ^c: p = 0.067|Δ0.9%^ ^d: p = 0.035|Δ0.9%^	0.49 ± 0.05 ^a: p = 0.011|Δ2.5%^ ^b: p = 0.067|Δ0.9%^ ^d: p = 0.062 || Δ0.0%^	0.50 ± 0.04 ^a: p = 0.008|Δ2.5%^ ^b: p = 0.067|Δ0.9%^ ^c: p = 0.062|Δ0.0%^	${x}^{2}$ = 17.505 P < 0.001	0.46 ± 0.04 ^b: p = 0.153|Δ4.3%^ ^c: p = 0.0119|Δ6.5%^ ^d: p = 0.042|Δ8.7%^	0.48 ± 0.03 ^a: p = 0.153|Δ4.3%^ ^c: p = 0.121|Δ2.1%^ ^d: p = 0.042|Δ4.2%^	0.49 ± 0.03 ^a: p = 0.119|Δ6.5%^ ^b: p = 0.121|Δ2.1%^ ^d: p = 0.071|Δ2.0%^	0.50 ± 0.03 ^a: p = 0.042|Δ8.7%^ ^b: p = 0.042|Δ4.2%^ ^c: p = 0.071 Δ2.0%^	${x}^{2}$ = 8.159 p = 0.043	Z = − 0.370 p = 0.711	Z = − 0.123 p = 0.902	Z = − 0.371 p = 0.710	Z = − 0.123 p = 0.902
ST10m (s)	1.90 ± 0.02 ^b: p = 0.005|Δ3.2%^ ^c: p = 0.005|Δ4.7%^ ^d: p = 0.005|Δ5.3%^	1.84 ± 0.02 ^a: p = 0.005|Δ3.2%^ ^c: p = 0.007|Δ1.6%^ ^d: p = 0.005|Δ2.2%^	1.81 ± 0.02 ^a: p = 0.005|Δ4.7%^ ^b: p = 0.007|Δ1.6%^ ^d: p = 0.012|Δ0.6%^	1.80 ± 0.02 ^a: p = 0.005|Δ5.3%^ ^b: p = 0.005|Δ2.2%^ ^c: p = 0.012|Δ0.6%^	${x}^{2}$ = 28.576 p < 0.001	1.89 ± 0.02 ^b: p = 0.007|Δ2.6%^ ^c: p = 0.007|Δ4.2%^ ^d: p = 0.008|Δ4.8%^	1.84 ± 0.03 ^a: p = 0.007|Δ2.6%^ ^c: p = 0.007|Δ1.6%^ ^d: p = 0.008|Δ2.2%^	1.81 ± 0.03 ^a: p = 0.007|Δ4.2%^ ^b: p = 0.007|Δ1.6%^ ^d: p = 0.031|Δ0.6%^	1.80 ± 0.02 ^a: p = 0.008|Δ4.8%^ ^b: p = 0.007|Δ2.2%^ ^c: p = 0.031|Δ0.6%^	${x}^{2}$ = 25.787 p < 0.001	Z = − 0.993 p = 0.321	Z = − 0.290 p = 0.772	Z = − 0.124 p = 0.901	Z = − 0.373 p = 0.709
ST20m (s)	3.22 ± 0.03 ^b: p = 0.005|Δ1.6%^ ^c: p = 0.005|Δ2.2%^ ^d: p = 0.005|Δ2.2%^	3.17 ± 0.03 ^a: p = 0.005|Δ1.6%^ ^c: p = 0.007|Δ0.6%^ ^d: p = 0.005|Δ0.6%^	3.15 ± 0.02 ^a: p = 0.005|Δ2.2%^ ^b: p = 0.007|Δ0.6%^ ^d: p = 0.011|Δ0.0%^	3.15 ± 0.02 ^a: p = 0.005|Δ2.2%^ ^b: p = 0.005|Δ0.6%^ ^c: p = 0.011|Δ0.0%^	${x}^{2}$ = 28.969 p < 0.001	3.23 ± 0.03 ^b: p = 0.007|Δ1.5%^ ^c: p = 0.007|Δ2.5%^ ^d: p = 0.008|Δ2.5%^	3.18 ± 0.02 ^a: p = 0.007|Δ1.5%^ ^c: p = 0.011|Δ0.9%^ ^d: p = 0.018|Δ0.9%^	3.15 ± 0.02 ^a: p = 0.007|Δ2.5%^ ^b: p = 0.011|Δ0.9%^ ^d: p = 0.257|Δ0.0%^	3.15 ± 0.02 ^a: p = 0.008|Δ2.5%^ ^b: p = 0.018|Δ0.9%^ ^c: p = 0.257|Δ0.0%^	${x}^{2}$ = 24.333 p < 0.001	Z = − 1.318 p = 0.188	Z = − 0.702 p = 0.482	Z = − 0.373 p = 0.709	Z = − 0.827 p = 0.408
ST30m (s)	4.37 ± 0.03 ^b: p = 0.005|Δ1.1%^ ^c: p = 0.005|Δ1.6%^ ^d: p = 0.004|Δ1.6%^	4.32 ± 0.03 ^a: p = 0.005|Δ1.1%^ ^c: p = 0.011|Δ0.5%^ ^d: p = 0.011|Δ0.5%^	4.30 ± 0.02 ^a: p = 0.005|Δ1.6%^ ^b: p = 0.011|Δ0.5%^ ^d: p = 0.480|Δ0.0%^	4.30 ± 0.02 ^a: p = 0.004|Δ1.6%^ ^b: p = 0.011|Δ0.5%^ ^c: p = 0.480|Δ0.0%^	${x}^{2}$ = 26.143 p < 0.001	4.37 ± 0.03 ^b: p = 0.007|Δ1.4%^ ^c: p = 0.006|Δ1.6%^ ^d: p = 0.007|Δ1.4%^	4.31 ± 0.03 ^a: p = 0.007|Δ1.4%^ ^c: p = 0.026|Δ0.2%^ ^d: p = 0.773|Δ0.0%^	4.30 ± 0.02 ^a: p = 0.006|Δ1.6%^ ^b: p = 0.026|Δ0.2%^ ^d: p = 0.005|Δ0.2%^	4.31 ± 0.02 ^a: p = 0.007|Δ1.4%^ ^b: p = 0.773|Δ0.0%^ ^c: p = 0.005|Δ0.2%^	${x}^{2}$ = 22.919 p < 0.001	Z = − 0.083 p = 0.934	Z = − 0.495 p = 0.621	Z = − 0.411 p = 0.681	Z = − 0.997 p = 0.319

HJ: horizontal-based jump group; VJ: vertical-based jump group; A1: assessment 1 (baseline); A2: assessment 2; A3: assessment 3; A4: assessment 4; ST: linear sprint test; 5-0-5: time at 5-0-5 test; BM: body mass; FM: fat mass; ^a^: significant different (p < 0.005) from A1; ^b^: significant different (p < 0.005) from A2; ^c^: significant different (p < 0.005) from A3; ^d^: significant different (p < 0.005) from A4 *significant different.

**Figure 5:**
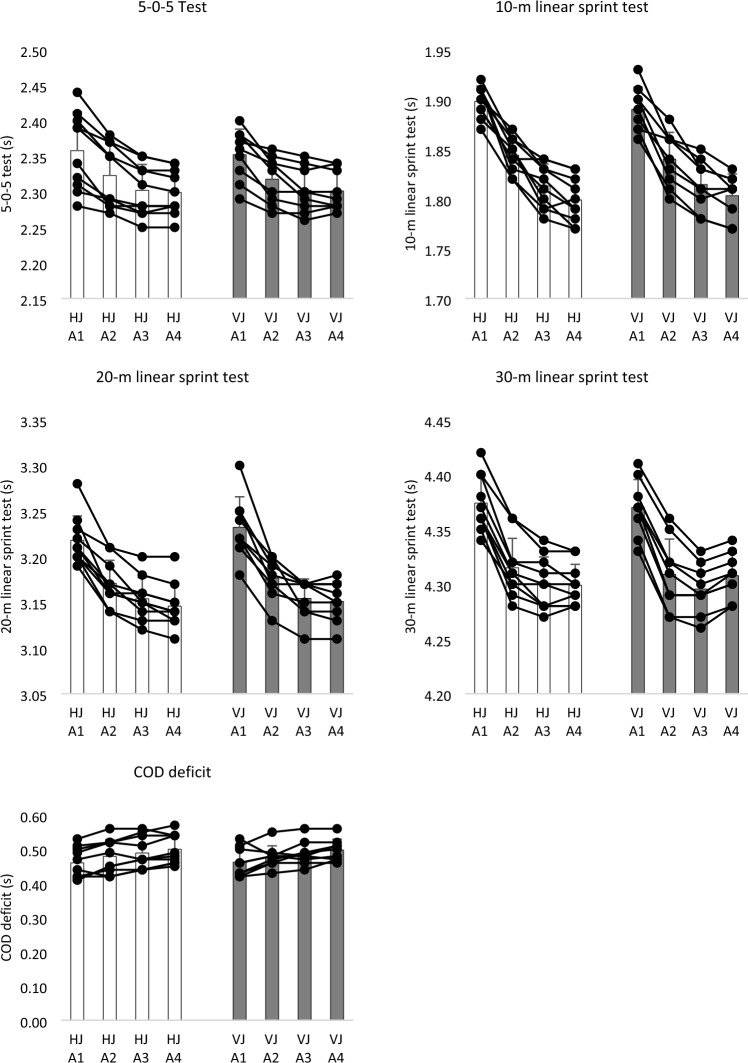
Descriptive statistics (mean and standard deviation) and intra-individual variations of players between assessment moments (A1, A2, A3, and A4) on 5-0-5 time, 10-m, 20-m and 30-m linear sprint tests in both horizontal jump training groups (HJ) and vertical jump training group (VJ).

Figure 5 presents the average and intra-individual variation of players’ change-of-direction and linear sprint performance over the assessments. Both groups significantly improved the 5-0-5 test from the first assessment to the second (p < 0.05) and from the second to the third (p < 0.05), although no significant changes were found from the third to the fourth assessment (p > 0.05). Considering the 10-m linear sprint time, both groups presented significant improvements from assessments 1 to 2 (p < 0.05), 2 to 3 (p < 0.05), and 3 to 4 (p < 0.05).

Significant improvements in 20-m linear sprint time were observed in both groups from the assessment 1 to 2 (p < 0.05), 2 to 3 (p < 0.05), and 3 to 4 (p < 0.05). Finally, significant improvements in 30-m linear sprint time were observed in both groups from the assessment 1 to 2 (p < 0.05) and 2 to 3 (p < 0.05). The HJ-group did not present significant differences from assessments 3 to 4 (p = 0.480). However, significant decrements were found from assessments 3 to 4 in VJ-group (p = 0.005).

## Discussion

This study is the first testing vertical vs. horizontal PJT conducted in soccer players lasting 21 weeks. This applied research allows an understanding of how coaches can use PJT over the season while integrating it into the periodization of the team. The current study shows that horizontal and vertical jump-oriented plyometrics produce similar beneficial effects on COD and sprinting of semi-professional soccer players.

Change-of-direction time, namely, the 5-0-5 test time, is nearly perfectly correlated with linear acceleration in small distances such as 5-, 10-m, and 20-m^29^. COD relies on the ability to accelerate and decelerate. Thus, this cohort's development of acceleration profiles from vertical and horizontal plyometric training appears favorable. Although we expected greater improvements from horizontal linear momentum force production to transfer to acceleration^30,31^, our results demonstrated that horizontal and vertical-oriented plyometrics produces similar effects for developing COD speed in semi-professional football players. Our results align with previous studies comparing horizontal and vertical plyometric jumping training^21,24^. Interestingly, no significant improvements were found between the last periods observed, which eventually can be associated with a plateau which is in line with some studies suggesting that no improvements occur at the mid and last third of the season^32^.

Although force-vector theory supports a hypothesis of a better transfer for specific movements (for example, vertical-oriented better for improving jump height, while horizontal-oriented better for acceleration and/or sprint)^33^, this has not been confirmed in original research using parallel-study designs^21,23,24^. For example, the linear sprint time observed in our study was significantly improved by horizontal and vertical-oriented plyometric jumps. It is plausible that the benefits of the improved stretch–shortening cycle and neural improvement for force production promoted by the plyometric training groups outweigh the anticipated direction-specific adaptations^34^.

The current study had some limitations. The first one is related to the small sample. However, as observed, the results were in line with previous findings that provide some confidence about the generalization of results. Another limitation is the absence of a passive control group, as data was collected during the regular training session. Future research should add this type of group to analyze if the changes in both groups are exclusively caused by plyometrics training or in combination with the regular training sessions.

While there are limitations, we emphasize the current study's practical implications, which suggest that a single training session of either horizontal or vertical oriented plyometrics training per week can significantly improve change-of-direction and linear sprints of semi-professional soccer players. Thus, coaches are free to select the exercises based on the most relevant factors since the effects seem similar in semi-professional soccer players.

## Conclusions

Our study revealed that horizontal and vertical-oriented plyometric jump training is similarly effective in improving semi-professional soccer players' change-of-direction and linear sprint. The improvements are observed over different periods, which suggests that the stimulus is effective for improving over a long period.

## Data Availability

The datasets used and/or analyzed during the current study are available from the corresponding author upon reasonable request.
